# Distinct impacts of heart rate and right atrial‐pacing on left atrial mechanical activation and optimal AV delay in CRT

**DOI:** 10.1111/pace.13401

**Published:** 2018-06-22

**Authors:** Andreas Kyriacou, Christopher A. Rajkumar, Punam A. Pabari, S.M. Afzal Sohaib, Keith Willson, Nicholas S. Peters, Phang B. Lim, Prapa Kanagaratnam, Alun D. Hughes, Jamil Mayet, Zachary I. Whinnett, Darrel P. Francis

**Affiliations:** ^1^ The Northern General Hospital Sheffield Teaching Hospitals NHS Foundation Trust Sheffield UK; ^2^ International Centre for Circulatory Health, National Heart and Lung Institute Imperial College London London W12 0HS UK

**Keywords:** atrioventricular delay, cardiac resynchronization therapy, electrophysiology‐clinical, heart failure, optimization

## Abstract

**Background:**

Controversy exists regarding how atrial activation mode and heart rate affect optimal atrioventricular (AV) delay in cardiac resynchronization therapy. We studied these questions using high‐reproducibility hemodynamic and echocardiographic measurements.

**Methods:**

Twenty patients were hemodynamically optimized using noninvasive beat‐to‐beat blood pressure at rest (62 ± 11 beats/min), during exercise (80 ± 6 beats/min), and at three atrially paced rates: 5, 25, and 45 beats/min above rest, denoted as A_paced,r+5_, A_paced,r+25_, and A_paced,r+45_, respectively. Left atrial myocardial motion and transmitral flow were timed echocardiographically.

**Results:**

During atrial sensing, raising heart rate shortened optimal AV delay by 25 ± 6 ms (P < 0.001). During atrial pacing, raising heart rate from A_paced,r+5_ to A_paced,r+25_ shortened it by 16 ± 6 ms; A_paced,r+45_ shortened it 17 ± 6 ms further (P < 0.001).

In comparison to atrial‐sensed activation, atrial pacing lengthened optimal AV delay by 76 ± 6 ms (P < 0.0001) at rest, and at ∼20 beats/min faster, by 85 ± 7 ms (P < 0.0001), 9 ± 4 ms more (P  =  0.017). Mechanically, atrial pacing delayed left atrial contraction by 63 ± 5 ms at rest and by 73 ± 5 ms (i.e., by 10 ± 5 ms more, P < 0.05) at ∼20 beats/min faster.

Raising atrial rate by exercise advanced left atrial contraction by 7 ± 2 ms (P  =  0.001). Raising it by atrial pacing did not (P  =  0.2).

**Conclusions:**

Hemodynamic optimal AV delay shortens with elevation of heart rate. It lengthens on switching from atrial‐sensed to atrial‐paced at the same rate, and echocardiography shows this sensed‐paced difference in optima results from a sensed‐paced difference in atrial electromechanical delay.

The reason for the widening of the sensed‐paced difference in AV optimum may be physiological stimuli (e.g., adrenergic drive) advancing left atrial contraction during exercise but not with fast atrial pacing.

## 1 INTRODUCTION

Optimization of atrioventricular (AV) delay has been shown to augment the acute hemodynamic benefits of cardiac resynchronization therapy (CRT) and has the potential to improve New York Heart Association (NYHA) functional classification and quality of life scores.^1^

At present, routine optimization of the AV delay is not universally performed. Where it is done, it is generally reserved for nonresponders of CRT and only carried out at rest. How the optimal AV delay changes with changes in heart rate and atrial pacing mode is not sufficiently understood in the CRT population.^2^

**Figure 1:**
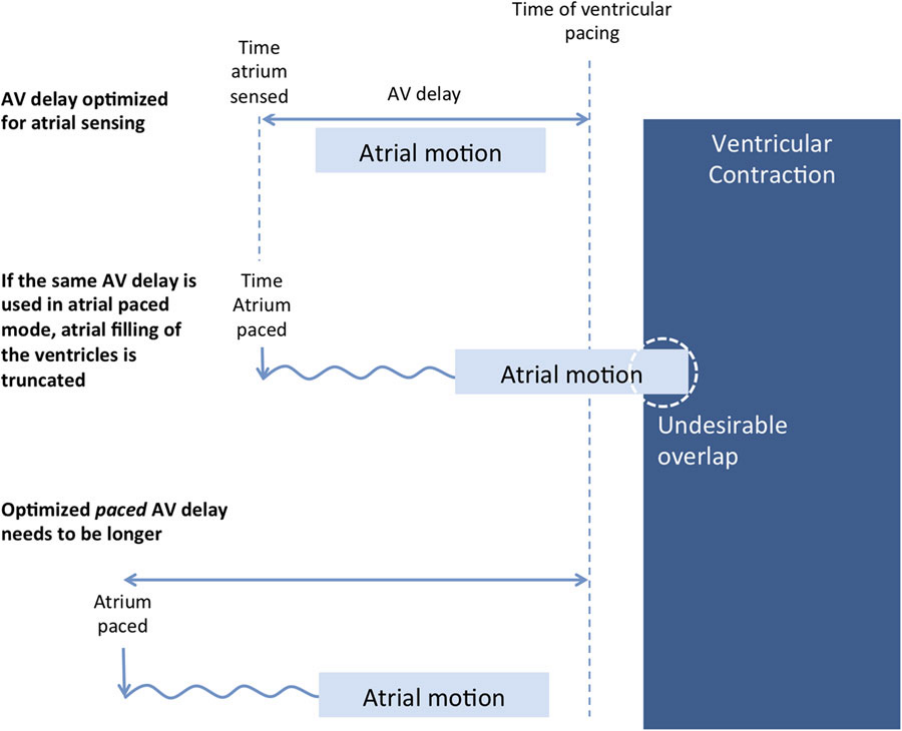
Schematic explanation of why a longer AV delay must be programmed for atrial paced than for atrial sensed to achieve the same time relationship between atrial and ventricular contraction. If hemodynamics are a result of motion of the myocardium, then it would be expected that the hemodynamically optimal programmed AV delays for sensed and paced atrial states would be providing broadly similar mechanical atrioventicular time intervals. AV = atrioventricular [Color figure can be viewed at http://wileyonlinelibrary.com]

In patients who have undergone physiological AV optimization with atrial sensing, the paced AV delay is commonly programmed longer by a fixed increment of 30 or 40 ms; this offset is applied across all heart rates. The principle behind this offset is to deliver the same time interval between mechanical motion of atrium and ventricle (Figure 1) regardless of whether the atrium is sensed or paced.

How much longer the delay must be to achieve the same timing relationship between atrial motion and ventricular activation, and whether this is different with changes in heart rate, has not been addressed with high precision measurements of myocardial motion. Nor has this been compared to high precision hemodynamic assessments. Most clinical research does not make enough measurements within individuals to present an error bar (precision estimate) within that individual. If the measurement is imprecise (e.g., vulnerable to biological variability), there is no possibility of showing association between different variables.^3^

Previous studies^4^, ^5^, ^6^, ^7^, ^8^ have used a wide range of cardiac response markers during AV optimization. However, many of these markers suffer from poor reproducibility, preventing precise identification of an optimal AV delay. As a result, it may not have been possible to identify underlying mechanisms responsible for changes in optimal AV delay.

In this study we assessed, with high precision measurements, the effect of atrial activation mode and heart rate on the atrial electromechanical delay and its relationship to the hemodynamically optimal AV delay.

## 2 METHODS

### 2.1 Subjects

Twenty patients (13 male, age 65 ± 8.7 years) were enrolled from heart failure and pacing outpatient clinics. All patients had a biventricular pacemaker or biventricular defibrillator in line with current guidelines (NYHA II‐IV despite maximal medical therapy and QRS > 120 ms). All subjects were recruited within 1 year of their CRT implantation date. Demographic characteristics of all subjects are included in Table 1. Exclusion criteria included the absence of sinus rhythm and the inability to exercise on a supine bicycle. Etiology of heart failure was ischemic in seven, dilated cardiomyopathy in 12, and hypertension in one. All subjects gave informed consent and the institutional review committee approved the study.

**Table 1:** Demographic characteristics of subjects

Demographic	n* *= 20
Gender	
Male	13
Age (years ± SD)	65 ± 8.7
NYHA functional class	
II (*n*)	14
III (*n*)	6
Aetiology of heart failure	
Ischemic	7
DCM	12
Hypertension	1
Baseline ECG parameters	
QRSd (ms)	158 ± 21
PR interval (ms)	189 ± 19
Echocardiographic measurements	
LVEDD (cm)	5.61 ± 0.89
LVESD (cm)	4.47 ± 0.93

*Note*: Data are means ± SD or number of participants. DCM = dilated cardiomyopathy; ECG = electrocardiogram; LVEDD = left ventricular end diastolic diameter. LVESD = left ventricular end systolic diameter; SD = standard deviation.

### 2.2 Study design and hemodynamic optimization

Hemodynamic optimization of the AV delay was carried out in five states (Table 2). Two states used atrial‐sensing mode and three rates used atrial pacing. In one state, A_sensed‐ex_, exercise was performed with a target heart rate of 20 beats/min above the resting sinus rate (r). Elevation of heart rate during exercise was achieved using a supine bicycle (Medical Positioning Inc., Kansas City, MO, USA). Patients were positioned supine during all five hemodynamic optimizations.

**Table 2:** Five pacing states at which hemodynamic optimization was performed

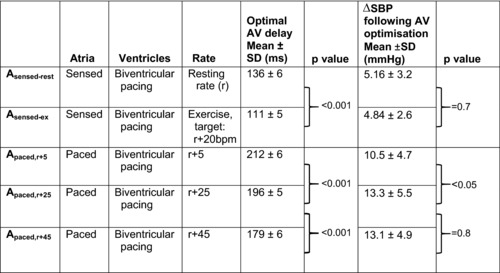

*Note*: Details of five pacing states used throughout the study protocol to examine changes in the hemodynamic optimal AV delay (“r” represents the patients resting sinus rate). Mean optimal AV delays at each pacing state are presented along with the mean increase in systolic blood pressure achieved with AV delay optimisation. AV = atrioventricular.

Our hemodynamic optimization protocol, performed at each pacing state, used an algorithm of repeated alternations from a reference AV delay of 120 ms and a tested AV delay. A range of AV delays were tested in a random order: 40, 80, 160, 200, 240, 280, and 320 ms, up to but excluding the AV delay at which atrioventricular conduction became solely intrinsic.

Our hemodynamic response marker used for optimization was change in systolic blood pressure (SBPrel) measured on a beat‐to‐beat basis by a Finometer (Finapres Medical Systems, Amsterdam, the Netherlands).^9^ This was placed between the proximal and distal interphalangeal joint of the middle finger on the right hand. Analog signals were taken via a National Instruments card (DAQ‐Card AI‐16E‐4, National Instruments, Austin, TX, USA) and Labview (National Instruments).

Change in SBP was calculated by subtracting the mean SBP of the beats approximating one respiratory cycle immediately following an AV delay transition from the mean SBP of the beats approximating one respiratory cycle immediately preceding the AV delay transition. We aimed for high reproducibility of optimization by averaging a minimum of 12 replicates of data, for each AV delay tested at each heart rate.^10^, ^11^, ^12^ We used parabolic interpolation to identify the optimal AV delay, defined as that which gave the greatest increase in SBP.^13^

Results were analyzed with custom software based on the Matlab platform (MathWorks, Natick, MA, USA).

### 2.3 Echocardiographic estimation of change in left atrial contraction timing

Echocardiographic images were acquired using a Vivid I system (GE Healthcare, Waukesha, WI, USA) with offline analysis using the Medcon software (McKesson, San Francisco, CA, USA).

Tissue Doppler imaging at the septal and lateral points of the mitral AV ring, and transmitral flow Doppler were acquired from the apical four‐chamber view. Images were acquired at four pacing states (A_sensed‐rest_, A_sensed‐ex_, A_paced,r+5_, A_paced,r+25_).

**Figure 2:**
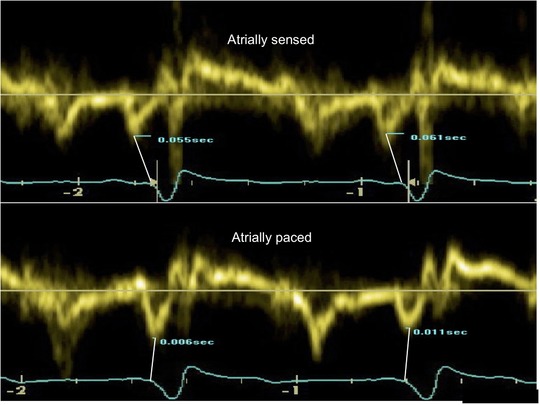
Example measurement of change in the timing of left atrial contraction when changing from a sensed to a paced atrium, at the same programmed AV delay. Tissue Doppler imaging across the septal wall of the mitral valve annulus, during sensed and paced atrial modes of biventricular pacing. The same AV delay was programmed for both modes. The time of contraction of the left atrium (peak of A’) was measured from a fixed time point (the onset of the QRS) in order to allow precise measurements of alterations in the time of left atrial contraction as a result of pacing changes. In this example, the A’ wave can be seen to be shifted to the right when atrially paced and this change denotes delay of left atrial contraction during atrial pacing. AV = atrioventricular [Color figure can be viewed at http://wileyonlinelibrary.com]

We measured the timing of left atrial contraction from the onset of QRS (the most reliably identifiable reference time point) during both atrial‐sensed and atrial‐paced measurements. For consistent identification of atrial contraction, we used the peak of the “A” wave on tissue Doppler, Figure 2 (at both the septal and lateral points of the AV ring) and also to the peak of the A wave on flow Doppler across the mitral valve, providing therefore three independent echocardiographic measurements of the change in the timing of left atrial contraction.

Ten consecutive beats (excluding ectopics) from the four‐chamber view were acquired and averaged, for each of the three echocardiographic measurements, at four pacing states, (A_sensed‐rest_, A_sensed‐ex_, A_paced,r+5_, A_paced,r+25_). All traces were recorded from the supine position. To make the comparisons fair, the AV delay was maintained at the same value at all pacing states. A detailed explanation of this echocardiographic parameter is provided in Appendix 1.

### 2.4 Statistical analysis

Paired comparisons of normally distributed continuous variables were made using Student's paired *t*‐test, and one‐way analysis of variance (ANOVA) where appropriate. A P‐value of < 0.05 was taken as statistically significant. Statview 5.0 (SAS Institute Inc., Cary, NC, USA) was used for statistical analysis.

## 3 RESULTS

The mean optimal AV delays and the associated increase in SBP achieved with AV optimization are presented in Table 2.

### 3.1 Effect of increasing heart rate on optimal AV delay

**Figure 3:**
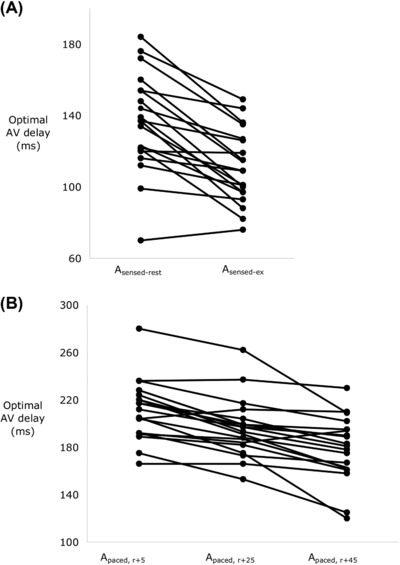
Individual patient data showing the effect of increased heat rate on the optimal AV delay. (A) During exercise to 18 ± 6 beats/min above the resting rate, there is a significant decrease in the hemodynamic optimal AV delay. (B) Pacing the atria to 20 (A_paced,r+25_) and 40 (A_paced,r+45_) beats above A_paced,r+5_, results in progressive shortening of the hemodynamic optimal AV delay. AV = atrioventricular

The optimal AV delay shortened from rest to exercise, Figure 3(A). Across all patients the optimal AV delay was 136 ± 6 ms at rest (A_sensed‐rest_) and 111 ± 5 ms during exercise (A_sensed‐ex_), P < 0.001.

Pacing the atria at incremental rates progressively shortened the optimal AV delay (Figure 3B) (F_ _= 8.31, P < 0.001): A_paced,r+5_ 212 ± 6 ms, A_paced,r+25_ 196 ± 5 ms (P < 0.001 vs A_paced,r+5_), and A_paced,r+45_ 179 ± 6 ms (P = 0.0001 vs A_paced,r+25_).

**Figure 4:**
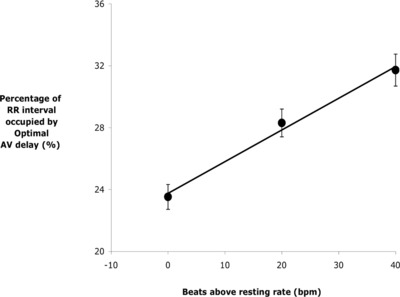
The effect of pacing the atria at incremental heart rates on the percentage of RR interval (cycle length) occupied by the optimal AV delay. With 20 beats/min increments in paced atrial rate, the optimal AV/RR ratio appears to increase linearly, r = 0.997, P < 0.05. AV = atrioventricular

During atrial pacing, the optimal AV delay expressed as a proportion of the RR interval increased with elevation of heart rate: 23.5 ± 0.8% at A_paced,r+5_, 28.3 ± 0.9% at A_paced,r+25_, and 31.7 ± 1% at A_paced,r+45_ (P < 0.00001, for all comparisons, Figure 4).

### 3.2 Sensed‐paced difference in AV delay that must be programmed to maintain optimal hemodynamics at the same heart rate

**Figure 5:**
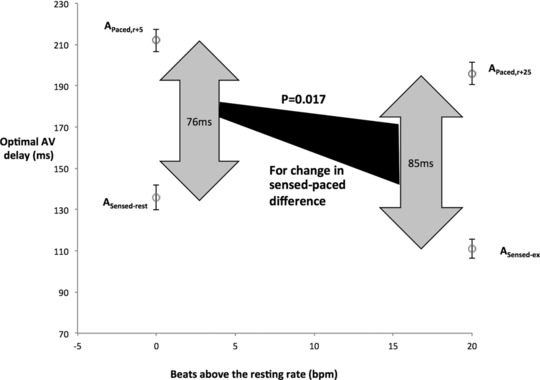
The sensed‐paced difference in hemodynamic optimal AV delay is larger at higher heart rate. The sensed‐paced difference in hemodynamic optimal AV delay is significantly greater at the higher heart rate, P = 0.017. AV = atrioventricular

In all patients, the hemodynamically optimal programmed AV delay found during atrial pacing was significantly longer than that during atrial sensing, at the equivalent heart rate (Figure 5). At A_sensed‐rest_, the optimal AV delay was 136 ± 6 ms versus 212 ± 6 ms (P < 0.0001) at A_paced,r+5_. During A_sensed‐ex_, the optimal AV delay was 111 ± 5 ms versus 196 ± 5 ms (P < 0.0001) at A_paced,r+25_.

The difference between sensed and paced hemodynamic optimal AV delays was slightly, but statistically significantly, greater at the higher heart rate; 76 ± 6 ms versus 85 ± 7 ms, P = 0.017 (Figure 5).

### 3.3 Mechanical basis for the dynamic sensed‐paced electrical difference of the optimal AV delay

**Timing of left atrial contraction**. Left atrial contraction was delayed when the atria were paced instead of sensed (Table 3). This delay was detected by all three echocardiographic markers used in this study, at both heart rates. For example, using septal tissue Doppler imaging (TDI), at rest the delay in left atrial contraction was 63 ± 5 ms and at 20 beats/min faster this delay between sensed‐paced atrium was larger at 73 ± 5 ms, P < 0.01.

**Table 3:** The delay of left atrial contraction due to atrial pacing using three echocardiographic parameters at rest and at 20 beats/min increment

Echo parameter	Sensed‐paced delay in left atrial contraction at rest (ms)	Sensed‐paced delay in left atrial contraction at rest +20 beats/min (ms)	P‐value
Septal TDI	63 ± 5.4	73 ± 5.4	<0.01
Lateral TDI	56 ± 6.0	67 ± 4.8	<0.05
Transmitral	56 ± 5.1	64 ± 5.1	<0.05
ANOVA P‐value	P = 0.61	P = 0.48	

*Note*: Note that as well as the increment in sensed‐paced delay between heart rates being statistically significant for each echo parameter, the increment is approximately the same for all three parameters even though they were measured independently from separately acquired data. This suggests that the change in sensed‐paced delay between heart rates is real rather than an artefact or noise. ANOVA = analysis of variance; TDI = tissue Doppler imaging.

Importantly, increasing heart rate by atrial pacing from A_paced,r+5_ to A_paced,r+25_ did not significantly change the timing of left atrial contraction. However, a rise of heart rate by exercise, A_sensed‐ex_, caused left atrial contraction to become earlier compared with A_sensed‐rest_ by 7 ± 2 ms (P = 0.001).

**Contraction *within* the left atrium**. The septal‐to‐lateral delay in left atrial contraction remained unchanged regardless of heart rate; A_sensed‐rest_ versus A_sensed‐ex_ (19 ± 5 ms vs 21 ± 5 ms, respectively, P = 0.7), and A_paced,r+5_ versus A_paced,r+25_ (12 ± 7 ms vs 15 ± 6 ms, respectively, P = 0.3).

The septal‐to‐lateral delay remained unchanged between sensed‐paced atrium; A_sensed‐rest_ versus A_paced,r+5_ (P = 0.17) and A_sensed‐ex_ versus A_paced,r+25_ (P = 0.16).

### 3.4 Evaluation of echocardiographic timing parameters

We compared the noise of the three echocardiographic parameters by comparing the standard errors of the mean of the measurements at A_sensed‐rest_ and A_paced,r+5_ modes. ANOVA demonstrated no superiority of any of the echo parameters: septal TDI 2.7 ms, lateral TDI 2.6 ms, and mitral valve flow 2.72 ms at A_sensed‐rest_ (F = 0.02, P = 0.98); septal TDI 2.6 ms, lateral TDI 3.0 ms, and mitral valve flow 2.8 ms at A_paced,r+5_ (F = 0.38, P = 0.69).

## 4 DISCUSSION

In this study, we found that the optimal AV delay shortens with increasing heart rate, regardless of whether the atrial rate is increased by exercise or by pacing.

Second, this study provides echocardiographic data for the mechanism behind the need for a longer AV delay to be programmed for atrial pacing than for atrial sensing. Independent investigation with Doppler echocardiography showed that a delay in the left atrial contraction from sensed to paced atrium is consistent with this being the cause. Measurements of the magnitude of this sensed‐paced difference in cardiac physiology were found to be concordant across three independent Doppler measures.

Finally, this sensed‐paced discrepancy in optimal AV delay is dynamic and enlarges at higher heart rates. This is because under atrial‐sensing, exercise causes left atrial contraction to occur earlier, while in contrast under atrial‐pacing, increasing the pacing rate does not advance left atrial contraction.

### 4.1 Effect of heart rate on optimal AV delay

This is the first study to explore the change in optimal AV delay of biventricular pacing at three atrially paced heart rates, in 20 beats/min increments, using high‐reproducibility hemodynamic measures. We found that the optimal AV delay shortens by on average 8 ms per 10 beats/min increase in heart rate. This slope is concordant with current device algorithms (based on bradypacing algorithms), which use slopes ranging from 5‐ to 10‐ms reduction in AV delay per 10 beats/min increase in heart rate.

In this study, we showed a positive correlation between an increase in heart rate and the optimal AV delay expressed as a proportion of the RR interval. With increasing heart rate, the optimal AV delay occupied a bigger proportion of the cardiac cycle (Figure 4). In other words, although the optimal paced AV delay does shorten with increasing heart rate, it shortens by a smaller proportion than the shortening of the RR interval.

It is plausible that in heart failure the duration of atrial contraction in diastole becomes more important at higher rates. At slow heart rates, most of the filling occurs by passive movement (transmitral E wave). As rate increases, the E and A transmitral filling waves would tend to merge: an adverse phenomenon which is diminished by the AV delay shortening in response. However, too much shortening would eventually cause truncation of the A wave which itself would impair filling. Optimal filling at higher heart rates therefore entails a trade‐off between E/A merging and A wave truncation. We found that the ratio between AV optimum/RR interval gives an insight to this balance.

This observation may be used clinically for time‐efficient optimization. By identifying the optimal AV delay at just one heart rate, the optimal AV delay can be predicted at other heart rates (Figure 4). This may offer a time‐efficient method to determine a hemodynamic optimal AV delay at rest, from data acquired at faster atrial‐paced rates where hemodynamic optimization is more precise.^10^

Rate adaptive AV delay is widely used in dual‐chamber pacing, where it has been shown to improve exercise tolerance and hemodynamic markers.^14^ Our results, showing a similar shortening of AV delay with increasing heart rates, suggest that it may be reasonable to extrapolate the benefits of rate adaptation of AV delay to the CRT population.

A combination of the rate‐response and rate‐adaptive functions has been reported to improve peak exercise heart rate, exercise time, METs, and VO2max in chronotropically incompetent patients who have a CRT device.^7^

Our data suggest that further studies are indicated to assess the short‐to‐medium term benefits on hemodynamics and functional capacity of the rate‐adaptive AV delay function in the CRT population.

### 4.2 Effect of heart rate on timing of left atrial contraction

Elevating heart rate by exercise caused an earlier left atrial contraction, i.e., shorter interatrial contraction time (IACT). However, elevation of heart rate using atrial pacing had no effect on IACT. The most likely explanation for the advanced left atrial contraction with exercise, but not pacing, is that sympathetic activation reduces IACT and/or atrial electromechanical coupling.

Intracardiac electrogram studies in patients with pacemakers for bradycardia indications have shown that increases in heart rate have no influence on IACT, in both atrially paced^15^ and atrially sensed modes.^16^, ^17^ An invasive study suggested that IACT is not changed by exercise in CRT patients.^6^

Our measurements combine interatrial conduction and electromechanical coupling times, to identify the AV delay that must be programmed for optimized hemodynamics. This could explain the differences from previous studies which focused solely on measuring electrical delays.

The absence of sympathetic discharge during atrial paced elevation of heart rate could explain the lack of an effect on IACT. An invasive study of IACT in CRT patients has also shown no effect of incremental atrial pacing on IACT, consistent with our findings.^18^

### 4.3 Effect of atrial pacing on timing of left atrial contraction

Left atrial contraction occurs later (in relation to the electrically marked time point on the pacemaker) when the atria are paced than when they are sensed. This delay became larger with increasing heart rate, as discussed above. The delay in left atrial contraction may be explained by three main factors.

First, there is a pause between the delivery of the pacing stimulus to the onset of right atrial capture—this delay is termed the “atrial pacing latency.” Second, right intra‐atrial depolarization time may be longer because depolarization begins outside the specialized fast atrial conduction pathways. Both of these factors contribute to a delayed depolarization wave reaching the interatrial connection bundles (such as Bachmann's bundle). Third, in atrial sensed mode, the CRT device only detects the intrinsic atrial depolarization when it has already propagated from the sinoatrial node to the right atrial pacing electrode. This “atrial sensing latency” shortens the AV delay needing to be programmed to achieve a given mechanical AV delay.

Several invasive studies have demonstrated the dramatic increase in IACT with atrial pacing in dual‐chamber pacemakers^15^, ^17^ and in CRT devices.^18^

Although we did not measure IACT directly, we found that the delay in left atrial contraction (Table 3) is similar to (or even slightly greater than) the increase in IACT with atrial pacing reported by other studies (36 ms^18^ and 50 ms^17^).

### 4.4 Effect of atrial pacing on optimal AV delay

One can only program an electrical AV delay, but what matters to hemodynamics is the mechanical coupling of the left atrium and ventricle.^19^, ^20^ We found that pacing the atria significantly increased the optimal electrical AV delay. The majority of the increase in the optimal electrical AV delay was explained by the delay in the left atrial contraction time.

Several studies have reported the dependence of optimal AV delay on IACT in dual‐chamber pacemakers.^19^, ^21^ Invasive experiments^18^ report a high correlation between paced and sensed IACT and the corresponding optimal AV delays in CRT devices.

### 4.5 Study limitations

In our study, the exercise increase in heart rate was only of ∼20 beats/min, while the pacing‐mediated increase was done at both ∼20 and ∼40 beats/min. This was because our preparatory work showed that it was challenging for patients to sustain the level of exercise needed for ∼20 beats/min and unrealistic to ask them to sustain ∼40 beats/min, at a steady state for the duration required for the hemodynamic optimization and echocardiographic imaging.

Our hemodynamic measure was noninvasive beat‐to‐beat blood pressure, and not cardiac output. Therefore, patient‐specific factors affecting the peripheral vasculature may affect the results. However, throughout the experiment each patient retained the finometer cuff in the same position. Using blood pressure rather than cardiac output allows our protocol to minimize noise by focusing on relative changes in blood pressure between different AV delay states, with the transitions performed repeatedly to reduce the standard error. The choice of noninvasive blood pressure as the variable monitored makes it easy to acquire and process hundreds or thousands of heartbeats, and the sensor can easily be kept in position; this approach minimizes the noise that cannot be helped from entering echocardiographic measurements because of the impracticability of keeping the probe in the correct position for hundreds or thousands of beats, and then measuring the traces. This approach of using pressure gives good reproducibility and concordance with invasive hemodynamic measures.^12^

Subjects were positioned supine for all aspects of the protocol so that postural effects on hemodynamics were minimized. However, as a result, we do not have data acquired in the upright posture.

## 5 CONCLUSION

High precision measurements show that the hemodynamically optimal AV delay of CRT devices shortens with elevation of heart rate. Separately, switching from atrial sensed to atrial paced at the same heart rate means that a longer AV delay needs to be programmed for optimal hemodynamics. The sensed‐paced difference in AV delay optimum widens at higher heart rates.

Three separate echocardiographic variables concordantly confirm that the mechanism for the sensed‐paced difference in AV optimum is the sensed‐paced difference in atrial electromechanical delay. The reason for its widening at higher heart rates appears to be additional physiological stimuli, such as autonomic tone and catecholamines, present during exercise but not present during fast atrial pacing. These findings have the potential to inform more effective optimization algorithms to augment the hemodynamic benefit of CRT.

## Supporting information

Supporting Information
