# Supplementary material for: Distinct impacts of heart rate and right atrial‐pacing on left atrial mechanical activation and optimal AV delay in CRT
Source: Pacing Clin Electrophysiol. 2018 Jun 22;41(8):959–66. doi: 10.1111/pace.13401 (PMC6099378; doi:10.1111/pace.13401)
Supplement: Supplementary file 1 — Supporting Information [file PACE-41-959-s001.docx]

**Appendix 1**

**Rationale for echocardiographic parameter under examination**

In this study, we use echocardiography to identify changes in the timing of left atrial contraction brought about by changes in heart rate and pacing state. The onset of the QRS complex provided a fixed electrical reference time point in each patient (as the AV delay was constant for all echocardiographic assessments). However, we needed an echocardiographic fiducial point for the assessment of left atrial contraction which was available in every patient.

It was important that such a time point was not just always available but also had acceptable reproducibility. We considered the use of the onset or the peak of the A wave on tissue Doppler imaging and flow Doppler across the mitral valve.

On both counts, the peak of the A wave performed better than onset, and therefore this time point was taken forwards for use in our protocol. We measured the time from the peak of the A wave to the onset of the QRS complex and examined how this parameter was affected by changes in heart rate and pacing state. Below we explain why the peak of the A wave was used instead of the onset.

**Always available**

The A wave onset was not always available, because on many occasions there was fusion between the E and A waves.

One could take the trough between the E and the A as the onset, but quite clearly this does not represent the same point on the notional curve of atrial contribution as the onset of the A wave does when E and A are clearly distinct.

Even worse, when there is substantial fusion of E and A (see figure below), the trough between the E and A is very close to the peak of the A. One might at that stage be tempted to use the onset of the fused E-A wave but as soon as one does this one introduces a discontinuity because one is suddenly registering a far earlier time than one would be using the trough-between-E-and-A method.

Because our study did not need to measure the actual atrial contraction time, we could avoid falling into these traps by using a fiducial point that would be more universally available than the A wave onset. We only needed to have a consistent fiducial point against which to measure other timings and we therefore used the peak of the A wave.


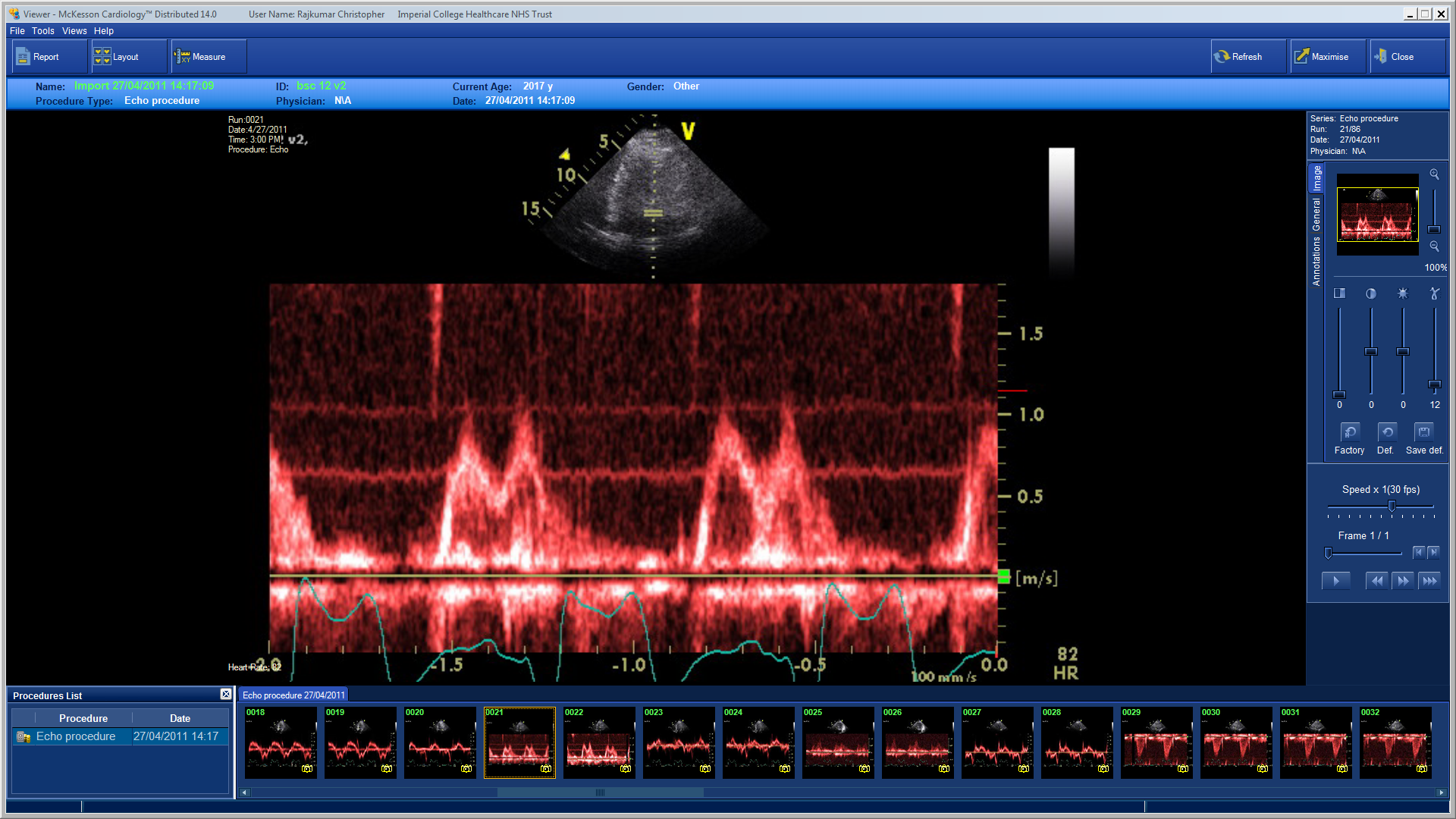


**Reproducible**

During the preparation of the study we assessed the inter-observer reproducibility and found it to be better with peak rather than onset of A wave (onset A wave SD 8.74ms, peak A wave SD 4.80ms; p = 0.03). The nature of sample size calculations is that we would have needed 4 times as much data to achieve as precise an answer with onset rather than peak, simply from this precision problem.

For these two reasons, we used peak of A wave rather than onset for the fiduciary point for timing. Readers should be aware that this is not the onset and therefore the time that follows should not be considered as the duration of atrial contraction.
